# High-Energy Computed Tomography as a Prospective Tool for In Situ Monitoring of Mass Transfer Processes inside High-Pressure Reactors—A Case Study on Ammonothermal Bulk Crystal Growth of Nitrides including GaN

**DOI:** 10.3390/ma15176165

**Published:** 2022-09-05

**Authors:** Saskia Schimmel, Michael Salamon, Daisuke Tomida, Steffen Neumeier, Tohru Ishiguro, Yoshio Honda, Shigefusa F. Chichibu, Hiroshi Amano

**Affiliations:** 1 Friedrich-Alexander-Universität Erlangen-Nürnberg, Crystal Growth Lab, Materials for Electronics and Energy Technology (i-MEET), Martensstraße 7, 91058 Erlangen, Germany; 2 Fraunhofer IIS, Fraunhofer Institute for Integrated Circuits IIS, Division Development Center X-ray Technology, 90768 Fürth, Germany; 3 Institute of Materials and Systems for Sustainability, Nagoya University, Nagoya 464-8601, Japan; 4 Friedrich-Alexander-Universität Erlangen-Nürnberg, Institute I: General Materials Properties, Martensstraße 5, 91058 Erlangen, Germany; 5 Institute of Multidisciplinary Research for Advanced Materials, Tohoku University, Sendai 980-8577, Japan

**Keywords:** X-ray computed tomography, gallium nitride, high-pressure reactor, in situ monitoring of crystal growth, ammonothermal growth of nitrides

## Abstract

For the fundamental understanding and the technological development of the ammonothermal method for the synthesis and crystal growth of nitrides, an in situ monitoring technique for tracking mass transport of the nitride throughout the entire autoclave volume is desirable. The feasibility of using high-energy computed tomography for this purpose was therefore evaluated using ex situ measurements. Acceleration voltages of 600 kV were estimated to yield suitable transparency in a lab-scale ammonothermal setup for GaN crystal growth designed for up to 300 MPa operating pressure. The total scan duration was estimated to be in the order of 20 to 40 min, which was sufficient given the comparatively slow crystal growth speed in ammonothermal growth. Even shorter scan durations or, alternatively, lower acceleration voltages for improved contrast or reduced X-ray shielding requirements, were estimated to be feasible in the case of ammonoacidic growth, as the lower pressure requirements for this process variant allow for thinned autoclave walls in an adapted setup designed for improved X-ray transparency. Promising nickel-base and cobalt-base alloys for applications in ammonothermal reactors with reduced X-ray absorption in relation to the maximum operating pressure were identified. The applicability for the validation of numerical simulations of the growth process of GaN, in addition to the applicability of the technique to further nitride materials, as well as larger reactors and bulk crystals, were evaluated.

## 1. Introduction

GaN is an important wide-bandgap semiconductor with applications ranging from optoelectronics to high-frequency and power electronics [1,2]. Benefitting from the small minority carrier diffusion length, the material has been successfully applied in energy-efficient blue and white light-emitting diodes, even when grown on foreign substrates [3]. However, it has become evident that, for further important applications in power electronics and lasers, low concentrations of structural defects are more crucial. Native substrates can strongly improve energy efficiency, reliability, and lifetime, especially for these types of GaN devices. This applies, in particular, to vertical devices [4]. Besides structural quality parameters, such as lattice curvature and control of impurity concentrations, cost is an important factor that partially determines the application space of GaN. One bulk growth method that combines high structural quality with very good scalability is the ammonothermal method [5]. Due to its scalability via the simultaneous growth of a large number of crystals, the ammonothermal method is viewed as a potential competitor for the currently more established hydride vapor phase epitaxy [2]. In addition, the ammonothermal method is increasingly used for the synthesis of further nitride materials, such as ZnGeN_2_ and InN [6,7,8]. To facilitate both scale-up of ammonothermal growth of GaN and the targeted application of ammonothermal growth to emerging nitride materials, an improved scientific understanding, in conjunction with the ability to perform sufficiently accurate numerical simulations of the growth process, is highly desirable. To judge the accuracy of numerical simulations, more comprehensive means of experimental validation are also highly desirable, as there are various potential sources of inaccuracy that remain difficult to quantify [9]. For both, the absence of a method to comprehensively track the mass transport of Ga during ammonothermal crystal growth remains an obstacle. Autoclaves equipped with ceramic windows, so-called optical cells, have been developed [10,11]. The use of ceramic windows enables optical access—e.g., for spectroscopic investigations—as well as access for medium-energy X-ray imaging [12,13]. The latter is capable of monitoring not only dimensional changes of GaN but also Ga concentration changes in the solution [14]. Nevertheless, the limited area of view inherent to the use of brittle windows in a pressure vessel (e.g., 6 mm window diameter for 300 MPa, 600 °C) does not allow for access to the entire autoclave volume [15]. A technique potentially capable of providing access to the entire autoclave volume is high-energy computed tomography (CT). In the field of crystal growth, this technique has already successfully been applied for evaluating the growth kinetics of SiC during bulk growth via the physical vapor transport (PVT) method [16]. For PVT growth of SiC, the applicability of computed tomography has been estimated to be practical for 6” or even 8” in tracking the movement of the growing crystal surface, though information on density variations inside the crystals (such as voids) is not obtainable at X-ray energies convenient for shielding [17]. Due to the somewhat high acceleration voltages and the continuous X-ray exposure in the order of minutes, the main application space of computed tomography in SiC is expected to be in research and development rather than in-line process monitoring [18].

In this work, a concept for in situ monitoring of Ga mass transfer throughout the autoclave volume was elaborated. Specifically, the potential of high-energy computed tomography for tracking of Ga mass transfer during ammonothermal growth was investigated.

A previous study has proven the feasibility in principle, albeit without a detailed analysis [19]. This contribution therefore provides a more comprehensive analysis that can guide the design of a respective experimental setup. Specifically, the spatial and temporal resolution and the feasibility of a setup for regular in-house use were investigated. Firstly, an experimental setup using existing components, suitable for ammonobasic growth, was analyzed. Secondly, a prospective setup with an autoclave construction optimized for X-ray transparency was investigated. The design for the autoclave with improved X-ray transparency assumed the use of ammonoacidic mineralizers. The reason was that these mineralizers enable the use of significantly lower pressures compared to ammonobasic mineralizers [20]. It should be noted that the mechanical considerations and resulting reactor designs were intended to evaluate the feasibility of X-ray computed tomography and to estimate suitable parameter ranges that can guide the dimensioning of the X-ray shielding components and the selection of CT components. These design considerations do not constitute a complete analysis from the perspective of operational safety, which is beyond the scope of this work. 

## 2. Materials and Methods

For the calculation of setup transmission as a function of X-ray energy, data on mass attenuation coefficients (total attenuation with coherent scattering) were obtained from the NIST database with an interval of 50 keV [21]. The compositions used for the calculations of X-ray transmission are listed in Table 1.

Data on the elemental composition and density of the alloys were taken from manufacturer datasheets [22,23,24,25,26,27,28,29,30,31]. Average values were used if ranges were given in the datasheets.

The remaining intensity after passing each setup component was calculated using the Lambert–Beer law and used as the initial intensity entering the next setup component. Calculations were undertaken as a function of photon energy. Note that CT uses continuous X-rays (emission spectrum of the anode material of the X-ray tube)—i.e., a spectrum consisting of a broad energy distribution—and the photon energy corresponding to the acceleration voltage represents the high energy limit of this spectrum. 

For the ex situ computed tomography measurements, a Makro-CT system developed by Fraunhofer EZRT was used. The main components of this system were a Comet X-ray source (600 kV, 0.5 mm focal spot size) and the Varex XRD1621 detector (2048 × 2048 pixels at 200 µm pixel size). Experimental parameters are listed in Table 2. 

## 3. Results and Discussion

**Figure 1:**
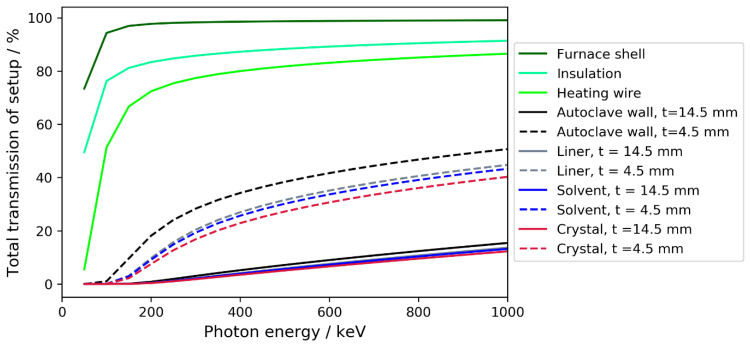
Calculated total X-ray transmission of an exemplary ammonothermal growth setup in two variants differing by the autoclave wall thickness *t* as a function of X-ray photon energy. The absence of data below 50 keV originates from calculating data at an interval of 50 keV starting at 50 keV. Total transmission would approach zero for photon energies approaching zero.

While lower X-ray energies generally improve image contrast and are easier to shield, it is also crucial to achieve sufficient X-ray transmission in the setup from X-ray source to detector. This is necessary for a sufficient signal-to-noise ratio and for reasonably short exposure times, which matter for the spatial resolution and temporal resolution, respectively. The decrease in the transmitted X-ray intensity with each setup component is shown in Figure 1, in which two variants of an ammonothermal growth setup are considered. A schematic of such a setup can be found in the literature, see Figure 3 in [9]. Components of the setup used in this study are listed in Table 3.

The two setup variants analyzed here differ in the autoclave wall thickness *t* and represent an experimentally verified design with *t* = 14.5 mm, as well as the thinnest wall thickness considered here of *t* = 4.5 mm. Due to their thickness, as well as elemental composition and high density, the nickel-base alloy autoclave walls play a dominating role in the overall absorption of an ammonothermal crystal growth setup. As evident from Figure 1, the considered reduction in the wall thickness has the potential to either significantly increase transmission, to significantly reduce X-ray photon energy, or to significantly reduce scan duration (exposure times). Of course, such a significant wall thickness reduction needs to be enabled by choosing a process variant that is capable of crystal growth at relatively low pressures. 

**Figure 2:**
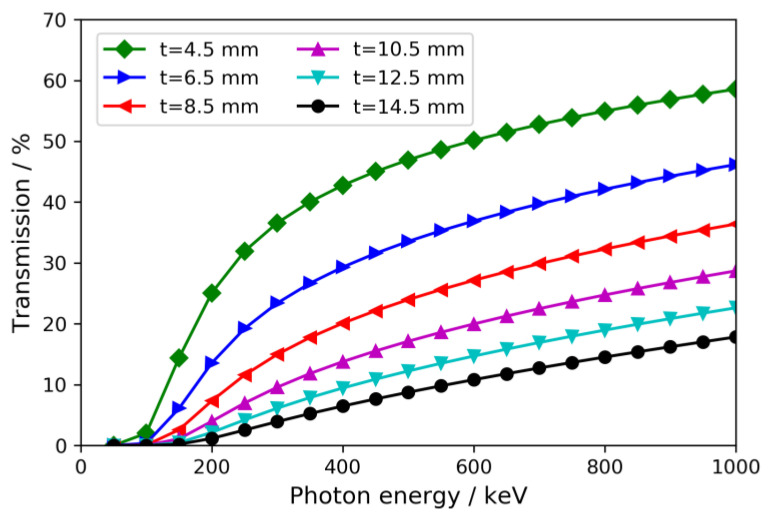
Calculated X-ray transmission of Inconel 718 for different values of the wall thickness *t* (i.e., a radiographed thickness of 2*t*) as a function of photon energy.

For the wall thicknesses experimentally investigated in this work, Figure 2 shows the autoclave wall transmission as a function of photon energy for the nickel-base alloy Inconel 718 [22]. See Section 2. Materials and Methods for methodical information on the calculations of X-ray transmission. Inconel 718 is one of the standard materials because it provides excellent strength, sufficient corrosion resistance for ammonobasic growth (depending on purity requirements), and good oxidation resistance at high temperatures, which permits use at ammonothermal growth temperatures in oxidizing environments (ambient air). The necessary wall thickness depends not only on the maximum operating parameters (pressure, temperature) of an autoclave but also on the mechanical properties of the alloy and their temperature dependence. 

**Figure 3:**
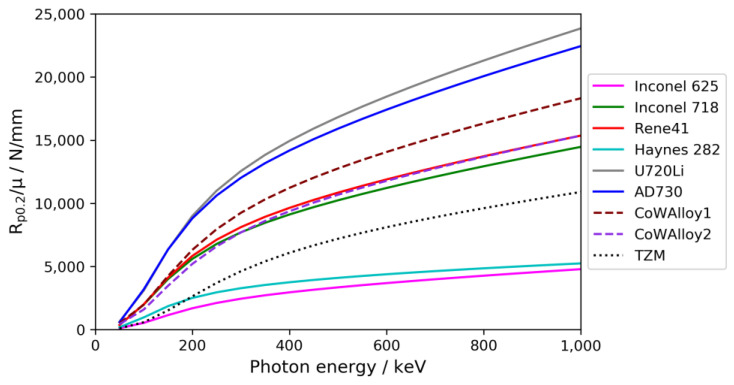
Calculated ratio of yield strength *R*_*p*0.2_ at 650 °C and linear attenuation coefficient *µ* as a function of photon energy for different alloys with high mechanical strength at high temperatures. Insofar as the alloys show significant precipitation hardening, the yield strengths of the precipitation-hardened condition were used. The absence of data below 50 keV is because data were calculated at an interval of 50 keV starting at 50 keV. The ratio *R*_*p*0.2_/*µ* would approach zero for photon energies approaching zero, as *µ* will become large for very low photon energies.

The X-ray transmission depends not only on the wall thickness but also on the alloy composition. To identify the best choice of alloy for a high-pressure reactor suited for in situ monitoring via X-ray CT, it is therefore reasonable to consider the ratio of yield strength *R*_*p*0.2_ at maximum operating temperature (value used: 650 °C) and linear mass attenuation coefficient *µ*. In Figure 3, this ratio is plotted against photon energy for the reference Ni-base superalloy Inconel 718, as well as for a selection of other commercial or experimental high-temperature alloys. Several of these alloys have already been used as materials for ammonothermal pressure vessels; namely, the nickel-base superalloys Inconel 718 [11], Inconel 625 [32], Rene41 [33], and Haynes 282 [34] and the molybdenum-base alloy TZM [35]. The experimental cobalt-base superalloys CoWAlloy1 and CoWAlloy2 have been investigated regarding their chemical stability in supercritical ammonia solutions containing ammonium chloride mineralizer [36]. The commercial nickel-base superalloys U720Li (ATI720^TM^) [23] and AD730 [24] were additionally taken into consideration due to their favorable mechanical strength in combination with relatively low contents of elements with high mass attenuation coefficients. 

**Figure 4:**
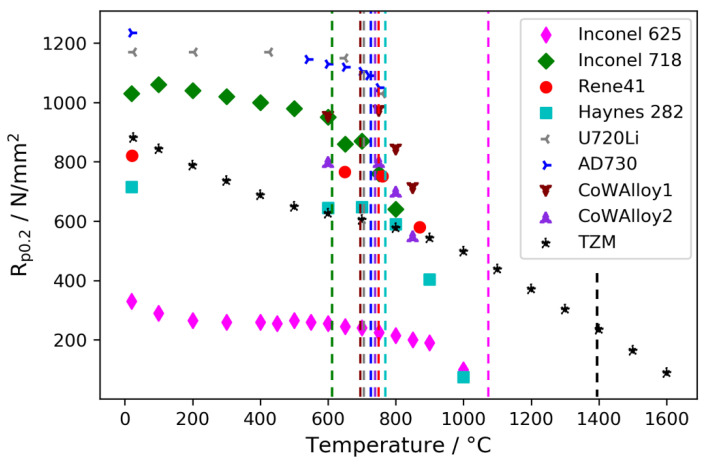
Yield strength of the different alloys as a function of temperature. The dashed lines represent the lowest temperature of the heat treatment.

It is evident from Figure 3 that the well-established nickel-base alloys Rene41 and Inconel 718 (2.4668-NiCr19Fe19Nb5Mo3) both provide a relatively high ratio of yield strength and linear attenuation coefficient, while the likewise well-established nickel-base alloys Inconel 625 (2.4856-NiCr22Mo9Nb) and Haynes 282 are clearly disadvantageous in terms of this ratio. Rene41 and Inconel 718 are well-suited provided the following conditions are met: Firstly, the chosen growth process variant must permit sufficiently low temperatures so that the operating temperature does not act as an unintended heat treatment that would alter the mechanical properties. Secondly, the chosen growth process variant must function at pressure and temperature conditions that render the material’s yield strength sufficient at the maximum operating temperature. Thirdly, the available lot of material needs to exhibit sufficient impact strength to minimize the risk of brittle failure. Specific requirements can arise from legal regulations for pressure vessels, depending on the country of operation. Heat treatment temperatures as relevant mechanical properties of a selection of alloys are given in Table 4. The yield strength generally decreases at elevated temperatures, and respective data for the considered alloys are given in Figure 4. 

The Mo-based alloy TZM clearly maintains mechanical strength to the highest temperatures; however, the ratio of the yield strength and linear attenuation coefficient is relatively low (Figure 3). In addition, the brittle–ductile transition of TZM occurs at a rather high temperature of 100 to 150 °C [37]. This can raise safety concerns when handling pressure vessels at low temperatures. In addition, TZM possesses a very high thermal conductivity, which needs to be taken into account for appropriate furnace design [38]. Nevertheless, the material has successfully been used as load-bearing material for ammonothermal autoclaves with operating temperatures up to 900 °C [35]. In addition, the chemical stability of TZM makes it a promising candidate for liner-free ammonothermal syntheses of Indium-containing materials [39]. Of the considered alloys, Inconel 718 possesses the highest yield strength at moderate temperatures; however, the low onset of age hardening around 620 °C (Table 4) and the decrease in yield strength at higher temperatures limits the operating temperature to 600 °C or slightly above. Inconel 625 does not share this issue; however, it only provides a fraction of the mechanical strength of Inconel 718. Haynes 282 features an intermediate yield strength that is maintained at moderately higher temperatures; however, it shows high X-ray absorption in relation to its yield strength (Figure 3). The experimental cobalt-base superalloys CoWAlloy1 and CoWAlloy2, especially the former, appear promising in terms of the combination of yield strength at high temperatures and relatively low X-ray absorption. However, these alloys are at a relatively early development stage and lack commercial availability [28,29,40]. The widely used nickel-base alloy Rene41 appears to show the best combination of properties among the commercially available alloys that have already been used for ammonothermal reactors, if no temperatures above about 740 °C are required. The commercially available nickel-base alloys U720Li and AD730 (NiCr16Co9Mo3W3Ti3Al2) are also promising candidates for temperatures below 650 °C and 730 °C, respectively, though the availability of material with sufficient Charpy impact strength would need to be verified. Note that the chemical stability of these alloys in ammonothermal reaction media has not been investigated yet and a detailed consideration of possible manufacturing issues is also beyond the scope of this study. 

Since the pressure and the necessary corrosion protection measures differ significantly for ammonobasic and ammonoacidic mineralizers, the choice of mineralizer may provide a valuable degree of freedom for the design of an effective model system of ammonothermal GaN growth that allows for Ga mass transfer tracking in the entire main volume of the autoclave. Among acidic mineralizers, which enable growth at relatively low pressures, the mineralizer that provides the highest growth rates for a given pressure is NH_4_F [20]. Therefore, the choice of NH_4_F allows for the lowest thickness in the load-bearing autoclave walls. Due to the corrosiveness of acidic mineralizers towards nickel-base alloys, it is necessary to employ a noble metal liner for corrosion protection of the autoclave, especially if prolonged use is intended, as in the case of an in-house computed tomography setup for regular use. Silver is fully compatible with NH_4_F and a proven liner material [41]. In addition, silver features a significantly lower atomic number than other potential noble metal liner materials (platinum and gold) and, consequently, results in relatively moderate X-ray absorption for a given radiographed thickness. 

### 3.1. Evaluation of Suitable Energy Range for X-ray CT Monitoring of GaN Growth

**Figure 5:**
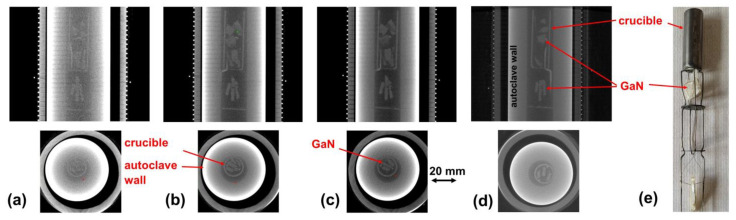
Computed tomography measurements obtained ex situ with an ammonothermal setup containing all typical elements except for the reaction medium. The material of the crucible was Inconel 718 in all cases. The material of the autoclave wall was Inconel 718 in (**a**–**c**), whereas an autoclave made of Haynes 282 was used in (**d**). Different acceleration voltages were tested: (**a**) 300 kV; (**b**) 550 kV; (**c**) 600 kV; (**d**) 590 kV. The autoclave wall thickness was *t* = 14.5 mm in all cases. Subfigure (**e**) shows a photograph of the setup placed inside the autoclave, consisting of the Inconel 718 crucible, Inconel 718 wire standoffs, and GaN crystal pieces hung using a molybdenum wire.

Computed tomography experiments conducted with existing ammonothermal autoclaves indicated that it is in principle feasible to monitor the distribution of GaN inside ammonothermal autoclaves even without adapted designs. In the case of an autoclave made of Inconel 718 designed for maximum operating conditions of 300 MPa and 600 °C, some transmission could be observed starting from about 300 kV, and a good level of transmission was obtained around 600 kV. Corresponding slices of CT reconstruction data are shown in Figure 5. As expected from the composition and density, a Haynes 282 autoclave of the same wall thickness (designed for 170 MPa, 800 °C) resulted in reduced transmission, though it was still possible to obtain evaluable images at 600 kV. The voxel size achieved within these measurements was 114 µm³, leading to a minimum detectable detail of approx. 228 µm³. Recent developments will make it possible to reduce the voxel size down to 50 µm³ and below at 450 kV in the future. To fully utilize the achievable voxel size within in situ measurements, movements of the crystals inside the autoclave may have an effect and should be avoided. This might require the application of advanced seed hanging methods. 

**Figure 6:**
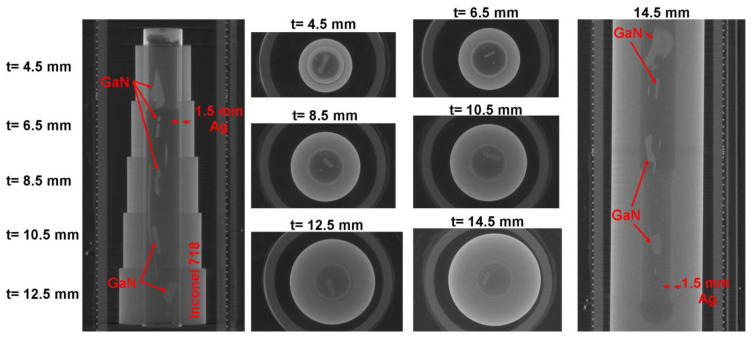
Ex situ computed tomography measurements obtained at 590 kV, 1 mA, and 2.5 s exposure time for each single projection image (all autoclave walls were Inconel 718). Left: vertical section through the setup with reduced autoclave wall thicknesses. Center: horizontal sections for each wall thickness. Right: vertical section of an established Inconel 718 autoclave designed for maximum operating conditions of 300 MPa and 600 °C. In the lower half, a 1.5 mm Ag tube shows the impact of a liner. The thin, lengthy, unlabeled objects in the vertical sections (also visible as a pair of dots in the horizontal sections) are Cu wires used as a standoff for hanging the GaN crystals.

The effects of a silver liner and of the thinning of the autoclave wall can be seen from Figure 6. As expected, any significant reduction in the autoclave wall thickness leads to noticeable improvements in the overall transparency of the setup. Likewise, a silver liner of 1.5 mm (2.7 mm in the uppermost part of the left subfigure of Figure 6) leads to a nonnegligible loss in transmission. An optimized setup should therefore reduce the radiographed thicknesses of both the autoclave wall and liner, as major absorbing components, to the minimum values deemed sufficient for the respective process variant and safety environment of the operation. 

### 3.2. Ammonobasic Growth of GaN

In the case of ammonobasic growth of GaN, pressures above 200 to 250 MPa are generally considered necessary, which is at least in part due to the onset of significant solubility in the GaN above this pressure range [42,43]. This leaves limited scope for reducing the autoclave wall thickness by limiting the maximum operating pressure, especially considering that a pressure range is needed for a growth process that needs to stay within a window of suitable parameters throughout the growth run. Pressure changes during growth can occur, amongst other reasons, due to the decomposition of ammonia [44]. Advantageously, ammonobasic solutions are much less corrosive to nickel-base alloys than ammonoacidic solutions, rendering the use of a liner optional unless especially high purity is required for the grown crystals [45,46]. Due to the high autoclave wall thickness, however, acceleration voltages around 600 kV can be estimated to be necessary obtaining obtain reasonable image quality within a scan duration of 20 min. Growth rates in the ammonobasic process variant as high as 344 µm/day have been reported [45]. Within 20 min, crystal growth may thus proceed up to 5 µm (2.5 µm on a single surface). Due to the achievable voxel size of 114 µm, the slight change in thickness during the scan will not affect the image quality of the CT at all. With the actual setup, two scans per day should be enough to adequately resolve the growth rate. 

A second matter of interest is monitoring of etch-back prior to growth, which is suspected to have a significant influence on the nucleation stage of crystal growth. Etch-back of GaN in ammonobasic media proceeds slowly compared to ammonoacidic experiments, at least in near-isothermal optical cell experiments in which convection does not play a major role for kinetics [42]. If etch-back leads to a significant dimensional change in the seeds, it is expected to be trackable by in situ CT. Moreover, it is expected that it will be possible to monitor the formation of parasitic deposition on reactor parts, which may help to prevent parasitic deposition and to better understand its effects on the growth on the seeds. Lastly, it is unknown if or in what way the nutrient undergoes morphological changes during the growth process. The nutrient constitutes a porous medium and its characteristics affect the flow field, temperature field, and, consequently, the supersaturation field. 

### 3.3. Ammonoacidic Growth of GaN

In the case of ammonoacidic growth of GaN, pressures around 100 MPa are sufficient [20]. Therefore, it is feasible to use lower autoclave wall thicknesses if a reactor is used only for ammonoacidic growth and specifically designed to minimize wall thickness. The permissible internal pressure *p* can be estimated from the yield strength *R*_*p*0.2_ and the ratio of the outer and inner diameter *u* as follows [47]:

(1) $$p=R_{p0.2}\frac{\ln\left(u\right)}{\sqrt{3}}$$

For instance, the yield strength of Inconel 718 at 650 °C is about 860 N/mm^2^; therefore, a pressure vessel with a maximum operating pressure of 120 MPa (safety factor of nearly 1.5; that is, with a permissible internal pressure of 177 MPa according to Equation (1)) could likely be designed with a wall thickness of 4.5 mm if assuming an inner diameter of 21 mm. This corresponds to the inner diameter of the autoclaves used in the test experiments and, specifically, to the thinnest test piece of Inconel 718 in Figure 6. However, age hardening in Inconel 718 begins at about 620 °C (Table 4), which raises doubts about the stability of its mechanical properties if exposed to temperatures above 620 °C for a prolonged time. The ammonoacidic growth using NH_4_F, which yields the highest growth rates at low pressures and is therefore of particular interest, is typically conducted in the retrograde solubility range at autoclave wall temperatures between 550 and 650 °C [20]. Therefore, Rene41 appears to be a superior choice, as it has significantly higher heat treatment temperatures, while its ratio of yield strength and linear attenuation coefficient is even slightly higher than that of Inconel 718 (see Figure 3). Further improvements may become possible pending the availability of alloys with improved properties, with cobalt-base alloys such as CoWAlloy1 showing promise. In addition, the use of a thinner Ag liner can be contemplated in a setup optimized for X-ray transparency. As a potential low-absorption material for corrosion protection, diamond stands out due to its abundant chemical stability in combination with its low X-ray absorption [48]. Recently, laminates of diamond foils with silver have been reported [49]. Such developments may also provide new possibilities for effective liner design with low X-ray absorption. 

For ammonoacidic growth using NH_4_F, growth rates of 410 and 465 μm/day in the c- and m-directions, respectively, have been reported [50]. As such, the scan duration used in the test experiments may be sufficient and it may be better to take advantage of the improved signal of an optimized setup. If, however, increased growth speeds or other fast processes should necessitate higher temporal resolution, the improved transmission of an optimized setup provides greater flexibility compared to one designed for the higher pressures of ammonobasic growth. Considering the calculated transmission of two exemplary setups that differed in the autoclave wall thickness and were very close to the Inconel 718 (*t* = 14.5 mm) setup in the test experiments, a reduction in the wall thickness from 14.5 mm to 4.5 mm yielded an improvement in overall setup transmission from 6.6 to 30.7% for X-ray photons of 600 keV. Therefore, with an optimized setup, the scan duration could likely be reduced to about one fifth; i.e., about 4 min. Etch-back of GaN in ammonoacidic ammonothermal solutions with NH_4_F is a considerably faster process compared to etch-back in ammonobasic solutions [12,14,42]. It is, therefore, a practical possibility that monitoring with a high frequency of scans of short durations would yield relevant insights during initial stages of the process, specifically during ramp-up of temperatures. Alternatively, a setup optimized for transparency would also make it possible to reduce the acceleration voltage considerably. For example, in the case of Inconel 718 with wall thickness reduced to 4.5 mm, 200 kV with a scan duration of about 20 min would yield a similar overall setup transmission as 600 kV with the same scan duration but a wall thickness of 14.5 mm (see Figure 1). Since lower photon energies generally improve the contrast in X-ray images, this is of interest both for the detection of small dimensional changes and potentially also for monitoring processes in the solution, such as changes in the concentration of Ga-carrying solutes. While the investigations reported here do not allow for a prediction of whether concentration changes of solutes would be detectable in CT measurements in windowless autoclaves, such changes have successfully been recorded in 2D X-ray imaging with pulsed exposures at 100 kV acceleration voltage in autoclaves with ceramic windows [14].

### 3.4. Applicability to Further Nitride Materials

**Figure 7:**
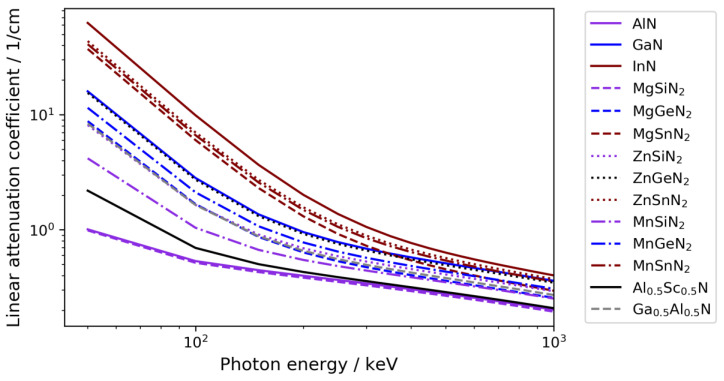
Linear attenuation coefficient of selected binary and ternary nitride materials. Data from the Photon Cross Sections Database XCOM of the National Institute of Standards and Technology (NIST) [21].

To evaluate the applicability of an ammonothermal high-energy CT to further materials, it is instructive to consider their linear attenuation coefficients, as the linear attenuation coefficient accounts for both the density and the elemental composition. Figure 7 shows the linear attenuation coefficient of selected emerging materials, as well as GaN for reference, as a function of photon energy. Of the shown materials, ZnGeN_2_ is practically equivalent to GaN in terms of its X-ray absorption. For the materials with higher linear attenuation coefficient—namely, InN, ZnSnN_2_, MnSnN_2_, and MgSnN_2_—even better contrast than shown for GaN is to be expected. Those with only moderately lower linear attenuation coefficients—namely, MnGeN_2_, MgGeN_2_, Ga_0.5_Al_0.5_N, ZnSiN_2_, and, possibly, MnSiN_2_—would likely still yield sufficient contrast, though the minimum detectable dimensional change may be higher than for GaN. For the representatives with the lowest X-ray attenuation coefficients—namely, Al_0.5_Sc_0.5_N, AlN, and MgSiN_2_—further investigations would be necessary, and the feasibility would also depend on the conditions required for their ammonothermal synthesis, which are partially unknown to date. Regardless of the limitations of X-ray imaging techniques at the light element side, growth process development for such materials would likely benefit from the improved fundamental understanding gained from investigations into well-trackable materials. 

### 3.5. Prospects for the Validation and Advancement of Numerical Simulations

As elaborated above, in situ CT is expected to be capable of tracking the mass transfer of GaN throughout the inner volume of a lab-scale ammonothermal autoclave. While such data are of direct interest to experimentalists, they are also of great interest in the context of numerical simulations. The mass transfer (dissolution of the nutrient and growth of the crystals, as well as parasitic deposition, if applicable) is the result of all the underlying physics that a numerical simulation of the growth process needs to address, with the thermal field, fluid flow field, and supersaturation field being key components of a simulation. It therefore provides valuable feedback on the accuracy of a growth process simulation. Note that a crystal growth experiment without in situ monitoring of Ga mass transfer does not provide nearly as comprehensive information in the case of ammonothermal growth. Since growth takes place in a closed system without a controllable source of variation in dopant supply or incorporation efficiency, it would be extremely difficult to utilize doping striations as a means of tracking the growth interface post-run. By providing time-resolved data on the spatial distribution of GaN throughout the growth run, an in situ CT would not only provide a means of validation but also yield the first hints as to when significant deviations between experiment and numerical model develop. This information is expected to be instructive for the search for their cause, which would benefit from the combination with complementary in situ monitoring technologies. For instance, in situ CT is fully compatible with in situ measurements of fluid temperatures at selected probing locations. Such fluid temperature measurements can provide valuable additional information not only on the internal thermal field but also on the stability of fluid flow and on chemical reactions associated with enthalpy changes [51]. Note that, regardless of the specific in situ monitoring technology used, care needs to be taken to establish matching thermal boundary conditions in both the experiment and numerical model [52] in order to provide the best possible similarity between the validation experiments and numerical simulations.

### 3.6. Potential for Application to Larger Autoclaves and Larger GaN Crystals

**Figure 8:**
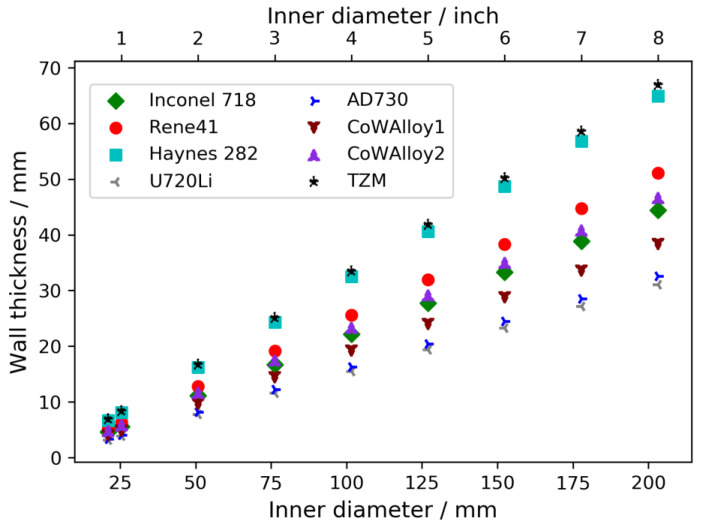
Wall thickness for a maximum operating pressure of 120 MPa estimated using Equation (1), with *R*_*p*0.2_ of the different alloys at 650 °C and a safety factor of 1.5 (i.e., *p* = 180 MPa). For better readability of the data for the remaining alloys, the values for Inconel 625 were excluded from the plot because the low mechanical strength would cause dissimilarly large wall thicknesses. The lowest inner diameter represents the experimentally studied geometry with 21 mm inner diameter.

**Figure 9:**
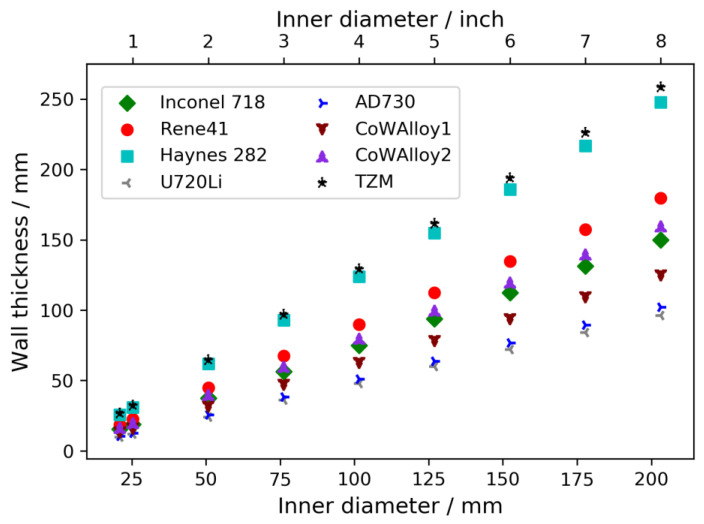
Wall thickness for a maximum operating pressure of 300 MPa estimated using Equation (1), with *R*_*p*0.2_ of the different alloys at 650 °C and a safety factor of 1.5 (i.e., *p* = 450 MPa). For better readability of the data for the remaining alloys, the values for Inconel 625 were excluded from the plot because the low mechanical strength would cause dissimilarly large wall thicknesses. The lowest inner diameter was represented by the experimentally studied geometry with 21 mm inner diameter.

Regarding the scalability of the technique, the following aspects need to be considered. Firstly, the growth of larger crystals or the simultaneous growth of a larger number of crystals requires the use of autoclaves with larger inner diameter. Based on Equation (1), the wall thickness of the autoclave increases for a given maximum operating pressure. For a maximum operating pressure of 120 MPa (ammonoacidic growth), estimates of the resulting wall thicknesses for different alloys are given in Figure 8, and Figure 9 presents the same information for a maximum operating pressure of 300 MPa (ammonobasic growth). 

As follows from Equation (1), the wall thickness increases linearly with increasing inner diameter and the slope depends on the yield strength of the load-bearing material, the maximum operating pressure, and the safety factor used. Consequently, alloys with high yield strength require comparably moderate increases in wall thickness. For instance, for a maximum operating pressure of 120 MPa, the wall thickness of an autoclave made from the nickel-base alloys U720Li or AD730 would be similar to that of the Inconel 718 autoclave that was experimentally investigated in this study. Therefore, it should be possible to apply high energy X-ray computed tomography to an autoclave with a 4” inner diameter, at least if the autoclave can be made from these materials. For larger autoclaves, the effectiveness of longer exposure times or, if unavoidable, higher acceleration voltages would need to be investigated. For maximum operating pressures of 300 MPa, only the applicability to research-scale autoclaves up to about 1” can reliably be judged on the grounds of the presented results. Clearly, for such high pressures, the required wall thicknesses are much higher and increase more severely with increasing inner diameter. Therefore, the process variants operating at moderate pressure (ammonoacidic process variants) have a fundamental advantage for in situ observation of mass transport in larger autoclaves. 

**Figure 10:**
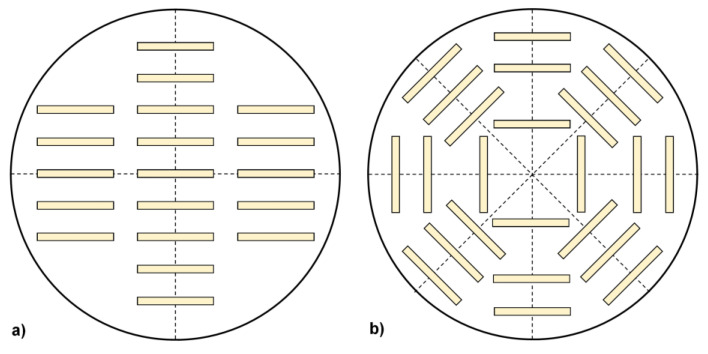
Considered options for the arrangement of seeds in a large reactor (top view of cross-section through the interior of an autoclave). Seeds are represented by yellowish rectangles. Dashed lines indicate mirror planes. (**a**) Example for conventional seed arrangement; (**b**) Proposed seed arrangement to reduce the length of radiographed path through GaN for improving X-ray transparency.

Secondly, the contribution of GaN crystals becomes significant for the overall transparency of the setup if the radiographed path within the crystals is long. This may apply to all or a subset of single exposures that are part of a CT scan, as each scan consists of many exposures that differ in the orientation of the setup in relation to the path of rays. Compared to a PVT growth of SiC [18], the simultaneous growth of many crystals with the ammonothermal method adds to the complexity of estimating the scalability of in situ monitoring via CT. In the example of SiC PVT growth, the information inside the crystals is lost eventually, though the outer dimensions can still be visualized (in analogy to a shadowgraph) [17]. In the case of the ammonothermal method, however, it would be particularly desirable to achieve total transmission because this would prevent seeds from blocking the X-rays from reaching inner seeds and exiting the setup thereafter. Therefore, it is expected that the scalability of the method will be best if the overall path of the rays through the GaN remains as short as possible for a given number and geometry of seeds. If seeds are placed without such considerations, large diameter seeds next to each other may cause a very long path for the rays in the in-plane direction. For example, consider a path for the rays from left to right in Figure 10a. By adjusting the arrangement of seeds, it is possible to decrease the maximum radiographed length within the GaN, without decreasing the number or diameter of seeds. See Figure 10b for an exemplary configuration that reduces X-ray absorption through an adapted arrangement of seeds. 

The suggested type of seed arrangement is also very interesting from the viewpoint of numerical simulations, as it is possible to increase symmetry. Such an arrangement may enable comparatively efficient 3D simulations by limiting the simulation domain to the smallest equivalent segment (such as one eighth in Figure 10b). While it is clear that, in reality, the flow will not always be perfectly identical in all segments (at least in a real, imperfect experimental setup), such a simulation in 3D but with reduced domain size might provide a compromise between 2D simulations and a 3D simulation of the entire autoclave volume. 

A third aspect to consider, especially upon scale-up, is the role of Bragg diffraction, which will be caused by the presence of large single crystals. Such effects were observed in the PVT growth of 3” SiC bulk crystals [17]. Depending on the energy of the diffracted radiation, it will be blocked by the autoclave walls and furnace to a smaller or larger extent. In the case of GaN, some Bragg reflections with high relative intensity are to be expected for the intense characteristic K_α_ radiation of a tungsten anode (as typically used in medium- and high-energy X-ray sources), corresponding to a photon energy of about 60 keV and diffraction angles of about 4 to 8 degrees [53]. Diffraction of X-rays with higher energies might also occur; however, higher energies will lead to a further compression of reciprocal space, leading to less pronounced deviations from diffraction-free penetration (which is normally assumed in CT reconstruction). Compared to SiC PVT growth, a fundamental difference in a scaled-up ammonothermal setup is that all or most crystals will change the positions of their centers upon rotation from one projection image to the next, further complicating possible effects of diffraction signals. While diffraction effects might lead to artifacts in CT reconstruction, there is also a possibility that such a diffraction signal could be evaluated as a measure of crystalline quality (see, for example, [10]). A comprehensive evaluation of useful or detrimental effects of diffraction in an ammonothermal CT would require respective experiments and is beyond the scope of the current study. 

A fourth aspect of scalability is the duration of the experiments, which tends to be larger if thicker crystals are to be grown. If reactors are exposed to high temperature under high mechanical stress for long cumulative durations (as determined by the number and duration of experiments, as well as the wall thickness and entire geometric design), they may experience more significant deformation effects from creep. This also depends on the creep mechanisms present in the respective alloy. In general, since the highest mechanical stresses in cylindrical high-pressure reactors tend to be located at the inner wall and in the gasket region, the established designs with thicker walls probably provide some protection against creep-induced deformation because the outer wall regions experience less stress and are therefore less affected by creep, providing a mechanical support to the region closer to the inner wall. While creep is a concern for the reactor lifetime, it is likely to first cause minor deformations in the gasket region, amongst others, which should cause leakages that can provide a hint that the condition of the reactor requires investigation. 

All in all, the effects of autoclave wall thickness, crystal absorption, and diffraction in a scaled-up ammonothermal reactor for CT demand further targeted investigation, especially if sufficiently transparent reactors can be realized using alloys with high mechanical strength and low X-ray absorption. For an assessment of reactor lifetime, possible effects of creep should be considered. 

## 4. Conclusions

High-energy computed tomography is a promising tool for in situ monitoring of mass transfer processes in lab-scale high-pressure reactors. For the ammonothermal growth of GaN, acceleration voltages in the range of 200 to 600 kV were estimated to be suitable to obtain results within a total scan duration of about 20 to 40 min, with suitable voltages depending on the process variant and its requirements for reactor wall thickness and reactor wall materials. With the commercial nickel-base alloys U720Li and AD730 and the experimental cobalt-base alloys CoWAlloy1 and CoWAlloy2, several alloys have been identified that hold the potential to realize reactors with reduced X-ray absorption by the autoclave walls beyond what is feasible with the established alloys so far used for ammonothermal reactors. This would also improve the prospects for applying the technique to larger reactors. 

While the technique possesses limited scalability due to the increase in reactor wall thickness with increasing inner diameters, it is well-suited for gaining fundamental insights via direct observation of the growth process in lab-scale autoclaves, as well as for the validation of numerical simulations. Well-validated numerical simulations can then facilitate the application of the obtained knowledge to larger systems, as the simulations do not have the same limitations in scalability. Realization of in situ CT measurements in ammonothermal crystallization processes is expected to contribute significantly to the fundamental understanding and development of accurate numerical simulations, which in turn are expected to facilitate efficient scale-up of ammonothermal crystal growth of GaN, as well as the targeted development of growth processes for emerging nitride materials. 

## Figures and Tables

**Table 1:** Compositions and density *ρ* of alloys used for calculations of X-ray transmission.

		Alloy								
		Inconel 718	Inconel 625	Rene41	Haynes 282	U720Li	AD730	CoWAlloy1	CoWAlloy2	TZM
*ρ*/g/cm^3^		8.260	8.440	8.249	8.280	8.090	8.230	8.830	8.900	10.220
Content/wt%	Ni	52.500	58.6000	49.2585	57.000	57.0610	58.1975	32.00	32.00	Zero
	Fe	35.765	5.000	5.0000	1.500	Zero	4.3000	Zero	Zero	Zero
	Cr	19.000	22.00	19.0000	20.000	16.5000	16.0000	12.00	12.00	0.1200
	Nb	5.125	1.750	Zero	Zero	Zero	1.1000	Zero	Zero	Zero
	Mo	3.050	9.00	9.7500	8.500	3.0000	3.0000	Zero	Zero	99.3714
	Co	1.000	1.000	11.0000	10.000	14.500	9.0000	42.30	40.80	0.4080
	Ti	0.900	Zero	3.1500	2.100	5.0750	3.6000	2.50	0.30	0.0030
	Al	0.500	0.40	1.6000	1.500	2.5500	2.2500	6.00	9.00	0.0900
	Mn	0.350	0.50	0.1000	0.300	Zero	Zero	Zero	Zero	Zero
	Si	0.350	Zero	0.5000	0.150	Zero	Zero	0.40	0.40	0.0040
	Cu	0.300	Zero	0.5000	Zero	Zero	Zero	Zero	Zero	Zero
	C	0.080	Zero	0.1200	0.060	0.0090	0.0100	0.08	0.08	0.0008
	Ta	0.050	1.750	Zero	Zero	Zero	Zero	1.50	0.20	0.0020
	P	0.015	Zero	Zero	Zero	Zero	Zero	Zero	Zero	Zero
	S	0.015	Zero	0.0150	Zero	Zero	Zero	Zero	Zero	Zero
	B	0.006	Zero	0.0065	0.005	0.0150	0.0125	0.08	0.08	0.0008
	W	Zero	Zero	Zero	Zero	1.2500	2.5000	3.00	5.00	Zero
	Hf	Zero	Zero	Zero	Zero	Zero	Zero	0.10	0.10	Zero
	Zr	Zero	Zero	Zero	Zero	0.0400	0.0300	0.01	0.01	Zero

**Table 2:** Experimental parameters of the ex situ CT measurements. The wall thickness *t* was 14.5 mm for the Inconel 718 and Haynes 282 autoclave. Thicknesses tested additionally using wall section samples were *t* = 4.5 mm, 6.5 mm, 8.5 mm, and 10.5 mm, respectively.

Autoclave	Number of Angular Steps	Exposure Time of Single Projection Image/ms	Scan Duration/min	Acceleration Voltages/kV
Inconel 718 autoclave	1200	1000	20	300; 550; 600
Inconel 718 wall sections	800	2500	33	590
Haynes 282	800	2500	33	590

**Table 3:** Components of the experimental setup considered for the calculation results presented in Figure 1. Minor simplifying assumptions used in the calculations can be seen from the comparison of the columns ”Material in Experiment” and “Material in Calculation”.

Component	Material in Experiment	Material in Calculation	Radiographed Thickness/mm
Furnace shell	Stainless steel	Fe	0.2
Insulation	47 wt% Al_2_O_3_, 53 wt% SiO_2_	47 wt% Al_2_O_3_, 53 wt% SiO_2_	80.0
Heating wire	Kanthal	Ni	1.0
Autoclave	Inconel 718	Inconel 718	14.5/4.5
Liner	Ag	Ag	2.0
Solvent	NH_3_, 233.95 kg/m^3^	NH_3_, 233.95 kg/m^3^	19.0
Crystal	GaN	GaN	2.0

**Table 4:** Relevant parameters of the considered high temperature alloys at 650 °C (unless otherwise stated). Mechanical properties refer to the solution-annealed/recrystallized and age-hardened condition for the precipitation-hardened alloys. The mechanical properties of TZM refer to the stress-relieved condition.

Alloy	*R*_*p*0.2_/N/mm^2^	Creep Limit *R*_*p*,0.1_/10^4^h/N/mm²	Rupture Elongation *A*/%	Heat Treatment Temperatures/°C
Inconel 718	860 [22]	370 [22]	12 [22]	Solution annealing: 940 to 1065 [22] Age hardening: 620 to 790 [22]
Inconel 625	245 [25]	215 [25]	35 (20 °C) [25]	Solution annealing: 1080 to 1160 [25] (no age hardening required) [25]
Rene41	765.3 [26]		14 [26]	Solution annealing: 1080 to 1177 [26] Age hardening: 760 to 899 [26]
Haynes 282	631 (sheet) [27]	545 (*R*_*p*,0.1_/10^3^) [27]	31 [27]	Solution annealing: 1121 to 1149 [27] Age hardening: 788 to 1010 [27]
U720Li	1170 [23]		8 [23]	Solution annealing: 1080 to 1100 [23] Age hardening: 650 [23]
AD730	1120 [24]		19 [24]	Sub-solvus heat treatment: 1070/1080 [24] Age hardening: 730/760 [24]
CoWAlloy1	975 [28]			Recrystallization: 1050 to 1075 Age hardening: 750 to 900
CoWAlloy2	825 [28]			Recrystallization: 1000 [29] Age hardening: 750 to 900 [29]
TZM	616 [30]	400 [30]		Recrystallization: 1400 [31]

## Data Availability

The data presented in this study are available upon reasonable request from the corresponding author.
